# Causal Relationships between Lipid-Lowering Drug Target and Aortic Disease and Calcific Aortic Valve Stenosis: A Two-Sample Mendelian Randomization

**DOI:** 10.31083/j.rcm2508292

**Published:** 2024-08-19

**Authors:** Liang Yang, Mingyuan Xu, Xixi Gao, Jingwen Liu, Dingkai Zhang, Zhaohua Zhang, Zhidong Ye, Jianyan Wen, Peng Liu

**Affiliations:** ^1^China-Japan Friendship Hospital (Institute of Clinical Medical Sciences), Chinese Academy of Medical Sciences & Peking Union Medical College, 100029 Beijing, China; ^2^Department of Cardiovascular Surgery, China-Japan Friendship Hospital, 100029 Beijing, China; ^3^Peking University China-Japan Friendship School of Clinical Medicine, 100029 Beijing, China

**Keywords:** Mendelian randomization, lipid-lowering drug target, aortic disease, calcific aortic valve stenosis

## Abstract

**Background:**

Proprotein convertase subtilisin/kexin type 9 (*PCSK9*), 3-hydroxy-3-methylglutaryl-coenzyme A reductase (*HMGCR*), cholesteryl ester transfer protein (*CETP*) and apolipoprotein C3 (*APOC3*) are pivotal regulators of lipid metabolism, with licensed drugs targeting these genes. The use of lipid-lowering therapy via the inhibition of these genes has demonstrated a reduction in the risk of cardiovascular disease. However, concerns persist regarding their potential long-term impact on aortic diseases and calcific aortic valve disease (CAVS). This study aims to investigate causal relationships between genetic variants resembling these genes and aortic disease, as well as calcific aortic valve disease using Mendelian randomization (MR).

**Methods:**

We conducted drug-target Mendelian randomization employing summary-level statistics of low-density lipoprotein cholesterol (LDL-C) to proxy the loss-of-function of *PCSK9*, *HMGCR*, *CETP* and *APOC3*. Subsequently, we investigated the association between drug-target genetic variants and calcific aortic valve stenosis and aortic diseases, including thoracic aortic aneurysm (TAA), abdominal aortic aneurysm (AAA), and aortic dissection (AD).

**Results:**

The genetically constructed variants mimicking lower LDL-C levels were associated with a decreased risk of coronary artery disease, validating their reliability. Notably, *HMGCR* inhibition exhibited a robust protective effect against TAA (odds ratio (OR): 0.556, 95% CI: 0.372–0.831, *p* = 0.004), AAA (OR: 0.202, 95% CI: 0.107–0.315, *p* = 4.84 × 10^-15^), and AD (OR: 0.217, 95% CI: 0.098–0.480, *p* = 0.0002). Similarly, *PCSK9*, *CETP* and *APOC3* inhibition proxies reduced the risk of AAA (OR: 0.595, 95% CI: 0.485–0.730, *p* = 6.75 × 10^-7^, OR: 0.127, 95% CI: 0.066–0.243, *p* = 4.42 × 10^-10^, and OR: 0.387, 95% CI: 0.182–0.824, *p* = 0.014, respectively) while showing a neutral impact on TAA and AD. Inhibition of *HMGCR*, *PCSK9*, and *APOC3* showed promising potential in preventing CAVS with odds ratios of 0.554 (OR: 0.554, 95% CI: 0.433–0.707, *p* = 2.27 × 10^-6^), 0.717 (95% CI: 0.635–0.810, *p* = 9.28 × 10^-8^), and 0.540 (95% CI: 0.351–0.829, *p* = 0.005), respectively. However, *CETP* inhibition did not demonstrate any significant benefits in preventing CAVS (95% CI: 0.704–1.544, *p* = 0.836). The consistency of these findings across various Mendelian randomization methods, accounting for different assumptions concerning genetic pleiotropy, enhances the causal inference.

**Conclusions:**

Our MR analysis reveals that genetic variants resembling statin administration are associated with a reduced risk of AAA, TAA, AD and CAVS. *HMGCR*, *PCSK9* and *APOC3* inhibitors but not *CETP* inhibitors have positive benefits of reduced CAVS. Notably, *PCSK9*, *CETP* and *APOC3* inhibitors exhibit a protective impact, primarily against AAA, with no discernible benefits extending to TAA or AD.

## 1. Introduction

Aortic diseases encompass a spectrum, including thoracic aortic aneurysm (TAA), abdominal aortic aneurysm (AAA), and acute aortic syndrome, with aortic dissection (AD) being the most prevalent and life-threatening form of acute aortic syndrome. The reported incidences of TAA and AD are approximately 5–10 per 100,000 and 2.6–3.5 per 100,000, respectively [1]. Population-based screening studies have estimated a varying prevalence of AAA, ranging from 1.9% to 18.5% in men and 0.1% to 1.2% in women [2]. Globally, the death rates attributed to aortic diseases have risen from 2.49 per 100,000 in 1990 to 2.78 per 100,000 in 2010 [3]. In 2019, a total of 172,000 deaths were attributed to aortic aneurysms [4], encompassing both TAA and AAA, reflecting a concerning trend driven by population growth and aging.

While aortic diseases exhibit distinct differences in terms of population prevalence, modes of inheritance, and predisposing genes, they also share common risk factors, including a family history of the disease, hypertension, smoking, and atherosclerosis [1, 5]. Notably, descending TAA and AAA are often associated with atherosclerosis, and over 30% of AD patients present with atherosclerosis [6]. Consequently, lipid-targeted therapy (statins) for atherosclerotic aortic aneurysms is recommended or considered reasonable in current guidelines due to its demonstrated benefits against adverse cardiovascular events [7, 8]. However, the question of whether statin therapy benefits individuals without atherosclerosis remains a subject of debate. Statins are also frequently prescribed to AD patients, although the supporting evidence is not robust [7, 9].

An increasing number of clinical studies are investigating the link between lipid levels, statin use, and aortic aneurysms. Meta-analyses of previous observational studies have suggested a protective effect of high-density lipoprotein cholesterol (HDL-C) on AAA, while the association with low-density lipoprotein cholesterol (LDL-C) is less clear [10, 11]. Nevertheless, these findings should be interpreted with caution as many of these studies did not account for statin use, which is prevalent in the population. Despite the uncertainty surrounding the association between lipid levels and AAA in observational research, statin use has consistently demonstrated a robust protective effect on AAA with reduced adverse outcomes [12, 13, 14]. In contrast, the beneficial impact of statins on TAA remains uncertain [15, 16, 17], and limited data have examined statin use specifically in aortic dissection. Moreover, while 3-hydroxy-3-methylglutaryl-coenzyme A reductase (*HMGCR*) has been the primary target for dyslipidemia therapy for many years, newer medications such as proprotein convertase subtilisin/kexin type 9 (*PCSK9*) inhibitors, cholesteryl ester transfer protein (*CETP*) inhibitors and apolipoprotein C3 (*APOC3*) inhibitors have emerged as novel targets for cholesterol-lowering drug development [18, 19, 20, 21]. Nevertheless, the effects of these newer medications on aortic diseases remain relatively unexplored.

Calcific aortic valve stenosis (CAVS) is the most frequent cause of aortic valve disease, and currently, there is no established pharmacotherapy to prevent aortic valve calcification [22]. Calcific aortic valve stenosis appears to be a multifactorial disease. Mechanically, lipid deposition accompanied by injury and inflammation initiates the process followed by osteogenic differentiation and calcification [22, 23]. Several observational studies have suggested a potential risk of aortic valve stenosis in individuals with dyslipidemia. However, randomized controlled trials have failed to demonstrate the benefits of statins in preventing the progression of aortic stenosis [24]. It is important to note that these trials were based on small sample sizes with short-term follow-up, and therefore might not have been able to show long-term benefits.

Mendelian randomization (MR) analysis is an emerging method that leverages genetic variants to investigate potential causal relationships between exposures and clinical outcomes. Unlike traditional observational studies, MR can mitigate ethical concerns, reduce costs, and account for confounding factors. Drug-target MR is a specialized form of MR that focuses on variants in or near genes encoding the drug target to create instruments that mimic a therapeutic intervention. Several drug-target MR studies have already demonstrated their reliability and concordance with clinical research [25, 26, 27]. Given the challenges associated with conducting large-scale randomized controlled trials to investigate the connection between lipid drug targets and aortic disease, MR represents a valuable alternative approach.

In summary, our hypothesis proposes that LDL-C-lowering drugs may possess causative protective effects against aortic diseases and CAVS. Consequently, this study employs drug-target MR to explore the genetic associations between lipid drug targets, namely *PCSK9*, *HMGCR*, *CETP*, and *APOC3*, and aortic diseases and CAVS.

## 2. Method

### 2.1 Data Sources 

This study relies on publicly accessible summary-level statistical data derived from genome-wide association studies (GWAS), predominantly involving individuals of European ancestry (Table 1). Summary statistics for LDL-C were sourced from the Global Lipids Genetics Consortium (GLGC), which conducted a case-control GWAS meta-analysis encompassing 173,082 individuals from 60 studies [28]. The coronary artery disease genome wide replication and meta-analysis (CARDIoGRAM) plus the coronary artery disease (C4D) genetics (CARDIoGRAMplusC4D) 1000 Genomes-based GWAS represents a meta-analysis of GWAS studies involving 60,801 cases of coronary artery disease and 123,504 controls [29]. Summary-level data of aortic diseases and CAVS were extracted from the latest R9 release of the FinnGen GWAS results [30]. In this updated release, data encompass 377,277 phenotyped and genotyped individuals, including 2272 clinical endpoints, with aortic aneurysms further classified into TAA and AAA. It is imperative to note that this study constitutes a secondary analysis of publicly available GWAS data, and all requisite ethical approvals were obtained in the primary reports.

**Table 1.** **Data source details**.

Phenotypes		Data source	N cases	Sample size	Population
Exposures					
	*PCSK9*	Global Lipids Genetics Consortium	NA	173,082	Predominant European
	*HMGCR*	Global Lipids Genetics Consortium	NA	173,082	Predominant European
	*CETP*	Global Lipids Genetics Consortium	NA	173,082	Predominant European
	*APOC3*	Global Lipids Genetics Consortium	NA	173,082	Predominant European
Outcomes					
	Coronary Artery Disease	CARDIoGRAMplusC4D Consortium	60,801	184,305	Predominant European
	Abdominal Aortic Aneurysm	FinnGen	3548	349,539	European
	Thoracic Aortic Aneurysm	FinnGen	3510	349,539	European
	Aortic Dissection	FinnGen	881	349,539	European
	Calcific Aortic Valve Stenosis	FinnGen	9870	402,311	European

*APOC3*, apolipoprotein C3; *CETP*, cholesteryl ester transfer protein; *HMGCR*, 3-hydroxy-3-methylglutaryl-coenzyme A reductase; *PCSK9*, proprotein convertase subtilisin/kexin type 9; CARDIoGRAMplusC4D, coronary artery disease genome wide replication and meta-analysis (CARDIoGRAM) plus the coronary artery disease (C4D) genetics; N cases, number of cases; NA, not available.

### 2.2 Study Design and Drug-target Instrument Selection

**Fig. 1.**
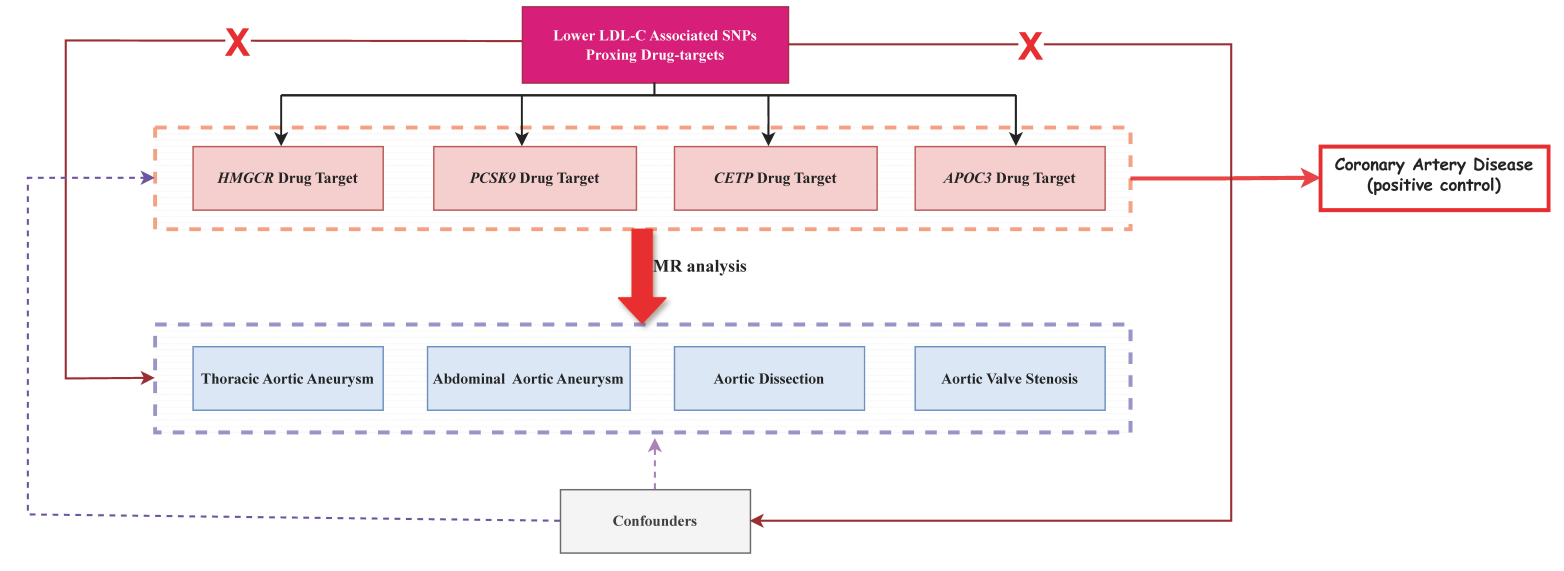
**Flowchart of the study. APOC3**, apolipoprotein C3; *CETP*, cholesteryl ester transfer protein; *HMGCR*, 3-hydroxy-3-methylglutaryl-coenzyme A reductase; LDL-C, low-density lipoprotein cholesterol; MR, Mendelian randomization; SNPs, single-nucleotide polymorphisms; *PCSK9*, proprotein convertase subtilisin/kexin type 9.

The overall study design is outlined in Fig. 1. In accordance with a previously reported approach [31], we identified single-nucleotide polymorphisms (SNPs) associated with lower LDL-C from the GLGC GWAS results at the genome-wide significance threshold of *p* < 5.0 × 10^-8^. SNPs located within ±100 kilobases of *PCSK9*, *HMGCR*, *CETP* and *APOC3* were designated as instruments and subjected to clumping at linkage disequilibrium (LD) r^2^ < 0.3 to serve as proxies for therapeutic intervention (see **Supplementary Table 1**). An F-statistic >10 denotes a robust correlation between the instrumental variables (IVs) and exposure. Given that *PCSK9*, *HMGCR*, *CETP* and *APOC3* inhibitors are commonly employed in the management of dyslipidemia and atherosclerotic cardiovascular disease, we used the CARDIoGRAMplusC4D 1000 Genomes-based GWAS results as a positive control to assess the validity of the constructed instruments. Subsequently, the identified drug-target instruments were harmonized with outcome datasets, and a two-sample MR analysis was conducted to examine the association between the constructed drug-target instruments and aortic disease, as well as CAVS.

### 2.3 Two-Sample MR Analysis

We utilized the “TwoSampleMR” and “MR-PRESSO” packages within R software (version 4.3.1, Lucent Technologies, Murray Hill, NJ, USA) to execute the MR analysis. Multiple MR analysis methods, including inverse variance weighting (IVW), median weighting, MR-Egger, and MR-pleiotropy residual sum and outlier (MR-PRESSO), were employed to explore causal relationships between drug-target instruments and aortic diseases, as well as CAVS. The IVW method furnishes a summary estimate of the causal effect of exposure on the outcome by amalgamating estimates from each SNP, with weights determined by the inverse of each instrument’s effect estimate variance [32]. Given its established reliability, IVW was designated as the primary method in this study. Median weighting, which assesses whether the majority of genetic variants are associated with the outcomes, was performed as part of sensitivity analysis [33]. We also incorporated the MR-Egger method, a modification of IVW that accounts for the presence of a non-zero intercept. If the intercept term equals zero precisely, the MR-Egger estimate aligns with the IVW estimate [34]. MR-PRESSO, meanwhile, was utilized to automatically detect potential outliers and remove SNPs exhibiting potential pleiotropy [35].

### 2.4 Quality Control and Sensitivity Analysis

To assess the significance and reliability of our results, several quality control measures were instituted. We employed a Bonferroni-corrected threshold of *p* < 0.0055 (0.05 divided by 3 exposures and 3 outcomes) to establish statistical significance. Nominal significant findings were identified using a threshold of *p* < 0.05. Cochran’s Q test was conducted to assess the heterogeneity of selected SNPs within the IVW model, with a *p*-value below 0.05 indicating potential heterogeneity. Furthermore, we implemented MR-Egger regression to identify and adjust for directional pleiotropy, with an unconstrained intercept. The leave-one-out function was executed to examine whether a single SNP disproportionately influenced the results and generated a forest plot for visualization purposes. We ascertained robust causal inferences by considering specific criteria based on our MR models and pleiotropy assessments [36]: (a) Consistent directional estimates were observed across various MR analysis methods. (b) The intercept term from MR-Egger regression exhibited no evidence of directional pleiotropy (*p* > 0.05). (c) Leave-one-out analysis revealed that a single SNP did not exert a substantial influence on the causal estimate.

## 3. Result

### 3.1 Drug-Target Instruments Selection

The particulars of the selected SNPs are delineated in **Supplementary Table 2**. A total of 28 SNPs were identified, corresponding to a one-unit standard deviation reduction in LDL-C levels achieved through pharmacological inhibition of *PCSK9*, *HMGCR*, *CETP* and *APOC3*. Among these, 13 SNPs were linked to *PCSK9*, 7 SNPs to *HMGCR*, 4 SNPs to *CETP* and 4 SNPs to *APOC3*. All SNPs exhibited F-statistics exceeding the threshold of 10, ranging from 24.55 to 650.06, signifying their robust representation of the target genes in the MR analysis. In line with our expectations, our genetically proxied instruments for *PCSK9*, *HMGCR*, *CETP* and *APOC3* in the Mendelian randomization analysis demonstrated a noteworthy reduction in susceptibility to coronary artery disease (see Table 2).

**Table 2.** **Relationship between constructed drug-target genetic variants and coronary artery disease**.

Outcome (positive control)	Drug Target	# of SNPs	Beta	SE	*p* value	OR (95% CI)
Coronary Artery Disease	P*CSK9*	13	–0.516	0.066	6.58 × 10^–⁢15^	0.597 (0.524–0.680)
Coronary Artery Disease	*HGMCR*	6	–0.353	0.080	1.12 × 10^–⁢5^	0.703 (0.600–0.822)
Coronary Artery Disease	*CETP*	4	–0.426	0.128	8.64 × 10^–⁢4^	0.653 (0.508–0.839)
Coronary Artery Disease	*APOC3*	4	–0.543	0.176	2.00 × 10^–⁢3^	0.581 (0.412–0.820)

*APOC3*, apolipoprotein C3; *CETP*, cholesteryl ester transfer protein; *HMGCR*, 3-hydroxy-3-methylglutaryl-coenzyme A reductase; # of SNPs, number of single-nucleotide polymorphisms; OR, odds ratio; *PCSK9*, proprotein convertase subtilisin/kexin type 9; SE, standard error.

### 3.2 Causal Inference of Drug-target Instruments and Aortic Disease and Calcific Aortic Valve Stenosis

**Fig. 2.**
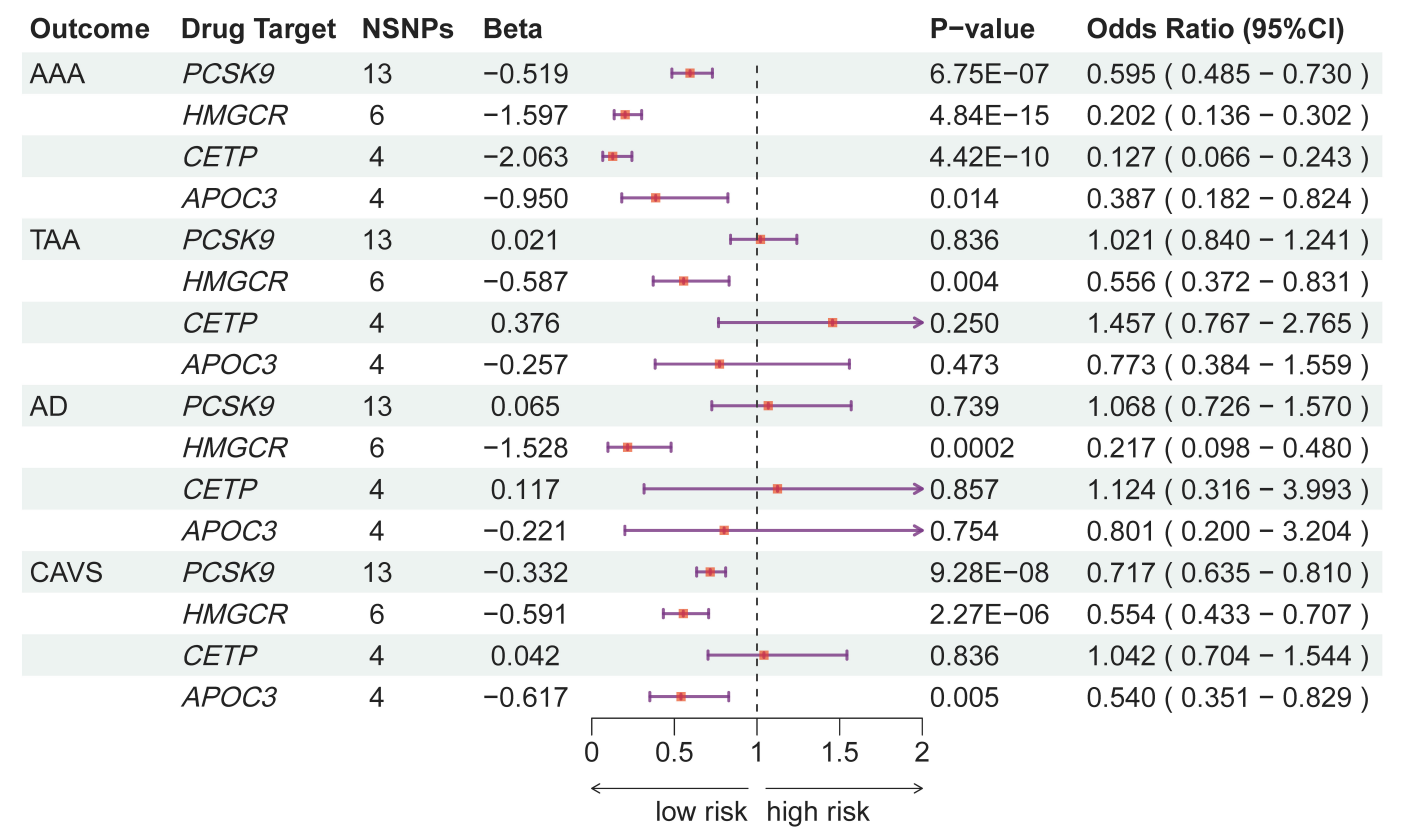
**Association of lipid drug targets with aortic disease and calcific aortic valve stenosis using the inverse variance weighted (IVW) method**. AAA, abdominal aortic aneurysm; AD, aortic dissection; *APOC3*, apolipoprotein C3, *CETP*, cholesteryl ester transfer protein; CAVS, calcific aortic valve stenosis; CI, confidence interval; *HMGCR*, 3-hydroxy-3-methylglutaryl-coenzyme A reductase; LDL-C, low-density lipoprotein cholesterol; SNPs, single-nucleotide polymorphisms; *PCSK9*, proprotein convertase subtilisin/kexin type 9; TAA, thoracic aortic aneurysm.

The comprehensive results are depicted in Fig. 2 and **Supplementary Table 3**. Initially, a conventional IVW MR analysis was conducted. The IVW model revealed that genetically constructed *PCSK9* inhibition significantly reduced the risk of AAA and CAVS (odds ratio (OR): 0.595, 95% CI: 0.4585–0.730, OR: 0.717, 95% CI: 0.635–0.810, respectively, *p* < 0.0055), whereas it did not yield a favorable outcome for TAA and AD. Similarly, targeted inhibition of *APOC3* was found to be associated with a reduced risk of AAA and CAVS (OR: 0.387, 95% CI: 0.182–0.824, *p* = 0.014; OR: 0.540, 95% CI: 0.351–0.829, *p* < 0.0055, respectively), while no protective effect was noted for TAA, AD, and CAVS. Proxied *CETP* inhibition was associated with a reduced risk of AAA (OR: 0.127, 95% CI: 0.066–0.243, *p* < 0.0055), but no evidence of a protective effect was observed for TAA, AD and CAVS. In contrast, the effect estimates for *HMGCR* inhibition were all statistically significant for all aortic diseases and CAVS, indicating a reduced risk: *HMGCR* inhibition and AAA (OR: 0.202, 95% CI: 0.107–0.315, *p* < 0.0055), *HMGCR* inhibition and TAA (OR: 0.556, 95% CI: 0.372–0.831, *p* < 0.0055), *HMGCR* inhibition and AD (OR: 0.217, 95% CI: 0.0.98–0.480, *p* < 0.0055), *HMGCR* inhibition and CAVS (OR:0.554, 95% CI: 0.433–0.707, *p* < 0.0055). It is noteworthy that one SNP (rs2006760) from the proxied *HMGCR* inhibition was excluded during harmonization with outcome data due to its palindromic nature with intermediate allele frequencies, resulting in the inclusion of 6 SNPs for MR analysis. To validate the IVW models further, MR-Egger regression, weighted median, and MR-PRESSO analyses were also conducted. In the statistically significant models mentioned above, the findings from these three analyses consistently aligned directionally with the IVW models, providing supplementary evidence for the robustness of the inferred causal association.

### 3.3 MR Sensitivity Analysis

The *p*-values of Cochran’s Q test, conducted between the four drug-target instruments and the four outcomes, were all below 0.05, signifying heterogeneity in the IVs (refer to Table 3). Except for *PCSK9*-AD, none of the MR-Egger regression intercepts deviated from zero (*p* > 0.05), indicating the absence of evidence for horizontal pleiotropy in the significant results. This enhances the validity of the causal inference drawn from the MR estimate. Additionally, the leave-one-out analysis confirmed that no individual instrumental variable unduly influenced the observed causal associations.

**Table 3.** **Heterogeneity and pleiotropy tests for associations of lipid drug targets with aortic disease and calcific aortic valve stenosis**.

MR analysis	# of SNPs	Heterogeneity test		Pleiotropy test		
		Cochran’s Q test	*p*-value	Egger intercept	SE	*p*-value
*PCSK9* - AAA	13	5.117	0.954	0.002	0.014	0.894
*HMGCR* - AAA	6	2.113	0.833	–0.013	0.017	0.871
*CETP* - AAA	4	2.108	0.550	–0.026	0.069	0.747
*APOC3* - AAA	4	3.501	0.321	0.069	0.130	0.647
*PCSK9* - TAA	13	8.564	0.740	0.015	0.013	0.275
*HMGCR* - TAA	6	3.403	0.638	–0.032	0.014	0.704
*CETP* - TAA	4	1.297	0.730	1.297	0.069	0.730
*APOC3* - TAA	4	1.239	0.744	–0.012	0.106	0.919
*PCSK9* - AD	13	10.054	0.611	0.057	0.026	0.047
*HMGCR* - AD	6	4.372	0.497	–0.115	0.028	0.496
*CETP* - AD	4	1.120	0.772	–0.032	0.136	0.837
*APOC3* - AD	4	2.700	0.440	–0.086	0.235	0.750
*PCSK9* -CAVS	13	2.268	0.999	–0.003	0.008	0.735
*HMGCR* - CAVS	6	2.295	0.807	–0.046	0.048	0.391
*CETP* - CAVS	4	0.990	0.804	0.040	0.042	0.443
*APOC3* - CAVS	4	0.517	0.915	–0.005	0.065	0.950

AAA, abdominal aortic aneurysm; AD, aortic dissection; *APOC3*, apolipoprotein C3; CAVS, calcific aortic valve stenosis; *CETP*, cholesteryl ester transfer protein; *HMGCR*, 3-hydroxy-3-methylglutaryl-coenzyme A reductase; MR, Mendelian randomization; # of SNPs, number of single-nucleotide polymorphisms; *PCSK9*, proprotein convertase subtilisin/kexin type 9; SE, standard error.

## 4. Discussion

Our rationale for selecting *PCSK9*, *HMGCR*, *CETP* and *APOC3* genes as focal points for investigation arises from the existence of licensed drugs that specifically target the pathways influenced by these genes. These therapeutic interventions include monoclonal antibodies and RNA interference targeting *PCSK9*, statins targeting *HMGCR*, obicetrapib for *CETP* inhibition and volanesorsen for *APOC3*. Several prior studies have utilized drug targets of *PCSK9* and *HMGCR* to explore their effects across diverse health domains, including cardiovascular diseases, diabetes, cancer, and neurocognitive function [25, 27, 37, 38]. Some of these investigations have yielded consistent results with clinical research and have even accurately anticipated the expected effects of therapeutic interventions using genetic instruments [39]. In the current study, we observed that combining multiple independently inherited common variants within or near the four genes associated with reduced LDL-C levels significantly lowered the risk of coronary artery disease. These findings lend support to the hypothesis that the identified genetic variants possess loss-of-function effects, reinforcing the rationale for drug-target inhibition.

Our drug-target MR analysis has unveiled compelling evidence regarding the protective effects of *HMGCR* inhibition against aortic diseases (AAA, TAA, and AD) and CAVS. Furthermore, our study has provided evidence of enduring genetic associations that emulate the potential long-term protective impact of *PCSK9*, *CETP* and *APOC3* inhibitors on AAA. In contrast, we have reported evidence indicating that the reduction of LDL-C with *PCSK9* and *CETP*-based therapeutic agents exerted a neutral influence on TAA and AD. Importantly, the drug-target inhibition of *HMGCR* exhibited a higher level of statistical significance, as evidenced by the *p*-values. Collectively, based on these results, we speculate with confidence that broad-spectrum lipid-lowering medications may mitigate the risk of AAA, and the frequent coexistence of AAA and atherosclerosis may help elucidate this association. It is noteworthy that, within this particular analysis, only *HMGCR* inhibition has demonstrated a potential reduction in the risk of TAA and AD.

The variability in the effects of different lipid-lowering medications on aortic disease, as observed in this study, remains incompletely understood. Nevertheless, a comprehensive and thoughtful analysis is likely to yield valuable insights into these variations. TAA and AD exhibit notable distinctions in their prevalence among populations, patterns of inheritance, and specific genes associated with predisposition when compared to AAA. At the cellular level, the distinct origins of smooth muscle cells in the thoracic aorta (neural crest and somitic mesoderm) compared to those in the abdominal aorta (splanchnic mesoderm) might contribute to different pathogenic mechanisms for AAA when contrasted with TAA and AD [40]. Approximately 20% of individuals affected by TAA or AD have a familial form of the condition characterized by an autosomal dominant pattern of inheritance, indicating that the development of thoracic aortic disease is often linked to mutations in a single gene, a phenomenon not typically observed in AAA [5]. Furthermore, it is worth highlighting that ascending TAA is frequently associated with connective tissue diseases, particularly Marfan syndrome, or a bicuspid aortic valve, further emphasizing the unique characteristics of the thoracic aorta [1]. Additionally, despite atherosclerosis being a shared risk factor for aortic diseases, its prevalence varies among populations. AAA is most commonly associated with atherosclerosis, followed by descending TAA, and finally ascending TAA and AD [6, 41]. One key aspect of treating atherosclerosis is lipid control, which may partially explain why lipid-lowering medications are more effective in managing AAA. By reducing lipid levels, these medications can decelerate the progression of atherosclerosis, subsequently reducing the likelihood of developing AAA.

Statins, which target *HMGCR*, have been widely used as a first-line therapy for lowering cholesterol levels and managing atherosclerotic cardiovascular disease over several decades. Previous studies have demonstrated that statins not only reduce perioperative morbidity and mortality rates during vascular surgery, including procedures for aortic aneurysms, but also decrease long-term adverse events and aneurysm growth in both AAA and TAA [8, 12, 15]. Initial speculations existed that TAA (especially ascending TAA) and AD might not benefit to the same extent from statin use as AAA, primarily because they are not closely associated with atherosclerosis. Conversely, our research findings suggest that statins have a protective effect on the thoracic aorta as well, which aligns with Angeloni’s report that statin treatment reduced the growth rate of ascending TAA and improved survival [8]. The pleiotropic effects of statin medications may elucidate why, among the selected lipid-lowering drugs, only statins exhibit a protective effect in various aortic diseases. Both *in vitro* and *in vivo* evidence suggest a correlation between statin therapy and reduced expression of metalloproteinases and proteolytic enzymes, whose upregulation promotes inflammation, leading to smooth muscle apoptosis [42]. Furthermore, statins effectively ameliorate endothelial dysfunction through pleiotropic actions, including increasing the expression of endothelial nitric oxide synthase (eNOS), promoting nitric oxide production, inhibiting Rho prenylation, and exerting antioxidant and anti-inflammatory effects [43, 44].

*PCSK9* inhibitors are emerging star drugs for regulating lipid levels associated with lower plasma LDL-C levels and a reduced risk of coronary heart disease. Phase 3 clinical trials have demonstrated their beneficial effects in treating high-cardiovascular-risk patients [45]. A recent genome-wide association meta-analysis, including the largest dataset from 14 discovery cohorts in the AAAgen Consortium, highlighted key mechanisms in AAA pathogenesis such as lipid metabolism, vascular development and remodeling, extracellular matrix dysregulation, and inflammation [46]. This study emphasized that a significant proportion of AAA loci likely functioned through modulating blood lipid levels, which in turn contributed to AAA development. It also suggested a genetic correlation between lipids and AAA, but not TAA, despite the genetic overlap between AAA and TAA. Further drug-target analyses indicated that higher genetically predicted circulating *PCSK9* and lipoprotein(a) were associated with an increased AAA risk, and a pre-clinical mouse model validated the important role of *PCSK9* in AAA development [46]. Consistent with this, our results also provide compelling evidence that genetic variants mimicking *PCSK9* loss of function significantly reduce the risk of AAA, while no beneficial effects were found against TAA and AD.

*CETP* and *APOC3* inhibitors represent promising therapeutic targets in the management of hyperlipidemia and atherosclerosis. Recent phase 3 and phase 2 clinical trials investigating *APOC* and *CETP* inhibitors have shown encouraging results, with reductions in plasma LDL-C levels and fewer adverse cardiovascular events [47, 48]. Our study provides robust evidence supporting a substantial reduction in the risk of AAA with the administration of both *APOC3* and *CETP* inhibitors. However, given the limited research in this area, it is important to interpret these MR results cautiously and further validate our findings through additional clinical studies. On the other hand, based on our study results, we do not recommend the use of *APOC3* and *CETP* inhibitors for the treatment of TAA and AD, as no beneficial outcomes were observed in these cases.

The present MR analysis reveals that inhibition of *HMGCR*, *PCSK9*, and *APOC3* has the potential to prevent CAVS, while *CETP* inhibition does not demonstrate any benefits in this regard. The CANHEART cohort study demonstrated an increased risk of severe aortic valve stenosis development in older adults with dyslipidemia (adjusted OR: 1.17, 95% CI: 1.14–1.21, *p* < 0.001) [49]. Furthermore, recent MR analysis suggested that lifelong exposure to high LDL-cholesterol levels increased the risk of symptomatic aortic stenosis, and LDL-lowering treatment might be effective in preventing its occurrence [24]. However, several randomized controlled trials have indicated that lipid-lowering therapy does not impede the progression of CAVS or reduce adverse aortic valve-related events [50, 51, 52]. The current MR analysis results also show conflicting results regarding the neutral impact of *CETP* inhibition on CAVS. While the use of Mendelian randomization is intriguing, its fallout may help elucidate isolated elements of disease mechanism, but certainly not the entire complexity of progressive valve disease [53]. Classic randomized controlled trials in humans remain the gold standard and the ultimate determinant for any intervention or medication intended to impede the progression to symptomatic aortic stenosis. Therefore, further exploration is required to determine whether lipid-lowering drugs have positive benefits against CAVS in the real world.

To the best of our knowledge, this is the first MR analysis that comprehensively investigates the causal relationship between lipid-drug targets and CAVS and aortic diseases, encompassing TAA, AAA, and AD. Previous MR studies have predominantly focused on the association of plasma lipid levels with AAA. For example, Harrison *et al*. [54] supported the hypothesis that lipids play a significant role in the development of AAA and suggested that LDL-C lowering drugs might serve as a potential treatment. However, their analysis did not include TAA and AD for further examination, and only 1-2 SNPs were used to proxy the drug target, which, as reported by Ference [55], may not provide quantitatively reliable estimates when relying solely on rare variants. Similarly, Chen *et al*. [56] and Li *et al*. [57] also demonstrated a causal relationship between plasma lipids and the risk of aortic aneurysms, highlighting the potential effectiveness of lipid drug targets in preventing and managing aortic aneurysms. However, their studies did not separately analyze TAA from other types of aortic aneurysms, which may introduce bias, as our current results suggest that TAA differs from AAA. In addition, a recent MR study explored the association between plasma lipids and the risk of aortic valve stenosis, revealing that lifelong exposure to high LDL-cholesterol increases the risk of symptomatic aortic stenosis [24]. However, this study did not further investigate the potential benefits of drug targets. Consequently, our study offers new insights into the causal relationship between lipid-drug targets and different types of aortic diseases, as well as CAVS.

In light of previous preclinical and clinical research, coupled with the findings of our current analysis, we recommend the widespread use of statins in the management of AAA. Furthermore, we advocate for further large-scale studies to investigate the potential benefits of statin therapy in the treatment of TAA and AD. Additionally, we suggest advancing clinical trials to assess the effectiveness of *PCSK9*, *CETP* and *APOC3* inhibitors in the treatment of AAA. Large-scale, randomized, controlled trials are needed to establish the role of different lipid-lowering drugs in patients with CAVS. These endeavors will contribute to expanding our knowledge and enhancing therapeutic approaches for aortic diseases.

## 5. Limitation

While MR analysis offers robust evidence that an exposure leads to an outcome while mitigating bias from confounding factors, it may not directly estimate the precise clinical benefits to be expected from a therapeutic intervention. Our study cannot address the question posed in the introduction regarding whether statin use or other lipid drugs benefit patients without atherosclerosis.

Furthermore, it is crucial to note that the dataset used in our study primarily consisted of individuals of European descent, which limits the generalizability of our findings to other ethnicities. Moreover, the publicly available data used in our study only provides summary-level statistics from whole-genome sequencing, preventing us from conducting subgroup analyses that adjust for factors such as gender and traditional risk factors (e.g., smoking, body mass index, and blood pressure). However, despite our efforts to address pleiotropy in our analyses, we acknowledge that pleiotropy remains a significant challenge in understanding the specific impact of lipid drug targets on aortic disease and CAVS.

## 6. Conclusions

Through Mendelian randomization analysis, we have uncovered a link between genetic variants resembling statin administration and a reduced risk of AAA, TAA, AD and CAVS. *HMGCR*, *PCSK9* and *APOC3* inhibitors but not *CETP* inhibitors have the positive benefits of reducing CAVS. Remarkably, *PCSK9* inhibitors, *CETP* inhibitors and *APOC3* inhibitors showed a protective effect specifically against AAA, while their beneficial effects did not extend to TAA or AD.

## Data Availability

The details of the data sources are provided in the methodology section, and they can be downloaded from the following websites: (1) http://csg.sph.umich.edu/willer/public/lipids2013/; (2) http://www.cardiogramplusc4d.org/data-downloads/; (3) https://finngen.gitbook.io/documentation/.

## Supplementary Material

Supplementary material associated with this article can be found, in the online version, at https://doi.org/10.31083/j.rcm2508292.
