# Supplementary material for: Causal Relationships between Lipid-Lowering Drug Target and Aortic Disease and Calcific Aortic Valve Stenosis: A Two-Sample Mendelian Randomization
Source: Rev Cardiovasc Med. 2024 Aug 19;25(8):292. doi: 10.31083/j.rcm2508292 (PMC11367000; doi:10.31083/j.rcm2508292)
Supplement: Supplementary file 1 [file 2153-8174-25-8-292-s1.docx]

Supplement Table 1. The location of drug target genes.

| Gene | hg19 | | | | hg38 | | | |
| --- | --- | --- | --- | --- | --- | --- | --- | --- |
|  | chromosome | location start | location end | ±100 kilobases | chromosome | location start | location end | ±100 kilobases |
| PCSK9 | 1 | 55505221 | 55530525 | 55405221-55630525 | 1 | 55039548 | 55064852 | 49939548-55164852 |
| HMGCR | 5 | 74632993 | 74657941 | 74532993-74757941 | 5 | 75336529 | 75362116 | 75236529-75462116 |
| CETP | 16 | 56995862 | 57017757 | 56895862-57117757 | 16 | 56961950 | 56983845 | 56861950-57083845 |
| APOC3 | 11 | 116700623 | 116703788 | 116600623-116803788 | 11 | 116829907 | 116833072 | 116729907-116933072 |

Hg19 and hg38 represents two versions assembly of GRCh37.p13 and GRCh38.p14, respectively; APOC3, apolipoprotein C3; CETP, cholesteryl ester transfer protein; HMGCR, 3-hydroxy-3-methylglutaryl-coenzyme A reductase; MR, Mendelian randomization; PSCK9, proprotein convertase subtilisin/kexin type 9.

Supplement Table 2. The selected SNP to proxy lipid drug targets.

| Drug Target  Gene | SNP | Chromosome | Position  (Hg19) | Position  (Hg38) | Effect Allele | Other Allele | Sample size | Beta | SE | *p* | EAF | R^2^ | F |
| --- | --- | --- | --- | --- | --- | --- | --- | --- | --- | --- | --- | --- | --- |
| *PCSK9* | rs2479394 | 1 | 55486064 | 55020391 | G | A | 172953 | 0.039 | 0.004 | 1.58 × 10^–19^ | 0.285 | 6.07 × 10^–04^ | 105.09 |
| *PCSK9* | rs11206510 | 1 | 55496039 | 55030366 | T | C | 172812 | 0.083 | 0.005 | 2.38 × 10^–53^ | 0.846 | 1.80 × 10^–03^ | 312.17 |
| *PCSK9* | rs2495495 | 1 | 55496556 | 55030883 | T | C | 162403 | 0.034 | 0.006 | 3.52 × 10^–08^ | 0.135 | 2.72 × 10^–04^ | 44.26 |
| *PCSK9* | rs2479409 | 1 | 55504650 | 55038977 | G | A | 172970 | 0.064 | 0.004 | 2.52 × 10^–50^ | 0.333 | 1.83 × 10^–03^ | 317.03 |
| *PCSK9* | rs11591147 | 1 | 55505647 | 55039974 | G | T | 77417 | 0.497 | 0.018 | 8.58 × 10^–143^ | 0.983 | 8.33 × 10^–03^ | 650.06 |
| *PCSK9* | rs4927193 | 1 | 55509872 | 55044199 | T | C | 173009 | 0.035 | 0.006 | 4.27 × 10^–11^ | 0.869 | 2.81 × 10^–04^ | 48.69 |
| *PCSK9* | rs11206514 | 1 | 55516004 | 55050331 | A | C | 172996 | 0.051 | 0.004 | 9.95 × 10^–33^ | 0.611 | 1.22 × 10^–03^ | 211.68 |
| *PCSK9* | rs572512 | 1 | 55517344 | 55051671 | T | C | 150564 | 0.048 | 0.005 | 5.31 × 10^–26^ | 0.346 | 1.03 × 10^–03^ | 155.76 |
| *PCSK9* | rs2495477 | 1 | 55518467 | 55052794 | T | C | 80151 | 0.064 | 0.005 | 7.29 × 10^–30^ | NA | 1.99 × 10^–03^ | 159.01 |
| *PCSK9* | rs585131 | 1 | 55524116 | 55058443 | T | C | 167769 | 0.064 | 0.005 | 2.70 × 10^–35^ | 0.815 | 1.22 × 10^–03^ | 205.27 |
| *PCSK9* | rs12067569 | 1 | 55528629 | 55062956 | A | G | 164264 | 0.089 | 0.010 | 1.97 × 10^–17^ | 0.034 | 5.19 × 10^–04^ | 85.27 |
| *PCSK9* | rs10493176 | 1 | 55538552 | 55072879 | T | G | 86056 | 0.078 | 0.010 | 2.54 × 10^–14^ | 0.885 | 1.22 × 10^–03^ | 105.45 |
| *PCSK9* | rs11583974 | 1 | 55551718 | 55086045 | A | G | 99955 | 0.065 | 0.012 | 3.95 × 10^–09^ | 0.030 | 2.46 × 10^–04^ | 24.55 |
| *HMGCR* | rs10066707 | 5 | 74560579 | 75264754 | A | G | 89888 | 0.050 | 0.005 | 2.97 × 10^–19^ | 0.417 | 1.20 × 10^–03^ | 108.08 |
| *HMGCR* | rs2006760 | 5 | 74562029 | 75266204 | G | C | 89885 | 0.053 | 0.008 | 1.67 × 10^–13^ | NA | 9.32 × 10^–04^ | 83.43 |
| *HMGCR* | rs72633962 | 5 | 74569028 | 75273203 | C | T | 83103 | 0.060 | 0.007 | 3.33 × 10^–15^ | 0.141 | 8.73 × 10^–04^ | 72.62 |
| *HMGCR* | rs3857388 | 5 | 74620377 | 75324552 | C | T | 172939 | 0.042 | 0.006 | 2.20 × 10^–11^ | 0.128 | 3.96 × 10^–04^ | 68.45 |
| *HMGCR* | rs10515198 | 5 | 74641560 | 75345735 | A | G | 173012 | 0.060 | 0.006 | 5.99 × 10^–22^ | 0.103 | 6.62 × 10^–04^ | 114.68 |
| *HMGCR* | rs12916 | 5 | 74656539 | 75360714 | C | T | 168357 | 0.073 | 0.004 | 7.79 × 10^–78^ | 0.431 | 2.64 × 10^–03^ | 444.94 |
| *HMGCR* | rs3804231 | 5 | 74696779 | 75400954 | A | G | 173020 | 0.064 | 0.005 | 1.88 × 10^–29^ | 0.132 | 9.44 × 10^–04^ | 163.46 |
| *CETP* | rs12448528 | 16 | 56985555 | 56951643 | A | G | 169282 | 0.037 | 0.005 | 1.06 × 10^–12^ | 0.227 | 4.80 × 10^–04^ | 81.34 |
| *CETP* | rs247616 | 16 | 56989590 | 56955678 | C | T | 171458 | 0.055 | 0.004 | 2.57 × 10^–37^ | 0.707 | 1.24 × 10^–03^ | 212.76 |
| *CETP* | rs12920974 | 16 | 56993025 | 56959113 | T | G | 82822 | 0.032 | 0.006 | 2.96 × 10^–08^ | 0.315 | 4.53 × 10^–04^ | 37.56 |
| *CETP* | rs1864163 | 16 | 56997233 | 56963321 | A | G | 171395 | 0.044 | 0.005 | 7.97 × 10^–21^ | 0.268 | 7.49 × 10^–04^ | 128.46 |
| *APOC3* | rs12272004 | 11 | 116603724 | 116733008 | A | C | 158514 | 0.068 | 0.008 | 8.24 × 10^–19^ | 0.061 | 5.26 × 10^–04^ | 83.37 |
| *APOC3* | rs11600380 | 11 | 116670182 | 116799466 | T | C | 139906 | 0.056 | 0.008 | 1.25 × 10^–11^ | 0.923 | 4.40 × 10^–04^ | 61.59 |
| *APOC3* | rs6589567 | 11 | 116670676 | 116799960 | A | C | 141691 | 0.049 | 0.006 | 3.12 × 10^–14^ | 0.137 | 5.75 × 10^–04^ | 81.58 |
| *APOC3* | rs10047462 | 11 | 116722041 | 116851325 | G | T | 171908 | 0.041 | 0.006 | 2.88 × 10^–10^ | 0.131 | 3.80 × 10^–04^ | 65.33 |

APOC3, apolipoprotein C3; CETP, cholesteryl ester transfer protein; EAF, effect allele frequency; HMGCR, 3-hydroxy-3-methylglutaryl-coenzyme A reductase; MR, Mendelian randomization; PSCK9, proprotein convertase subtilisin/kexin type 9; SNP, single-nucleotide polymorphism; SE, standard error.

Supplement Table 3. Correlated Mendelian randomization analysis results for drug targets on aortic disease and calcific aortic valve stenosis.

| Outcome | Drug target gene | Method | # of SNPs | Beta | SD | *p*-value | Odds Ratio | lower | higher | Confidence Interval |  |
| --- | --- | --- | --- | --- | --- | --- | --- | --- | --- | --- | --- |
| AAA | PCSK9 | Inverse Variance Weighted | 13 | –0.519 | 0.105 | 6.75 × 10^–07^ | 0.595 | 0.485 | 0.730 | 0.485–0.730 |  |
|  | PCSK9 | Weighted Median | 13 | –0.539 | 0.131 | 3.79 × 10^–05^ | 0.584 | 0.452 | 0.754 | 0.452–0.754 |  |
|  | PCSK9 | MR Egger | 13 | –0.535 | 0.158 | 0.006 | 0.585 | 0.430 | 0.797 | 0.430–0.797 |  |
|  | PCSK9 | MR Presso | 13 | –0.519 | 0.068 | 6.26 × 10^–06^ | 0.595 | 0.520 | 0.680 | 0.520–0.680 |  |
|  | HMGCR | Inverse Variance Weighted | 6 | –1.597 | 0.204 | 4.84 × 10^–15^ | 0.202 | 0.136 | 0.302 | 0.136–0.302 |  |
|  | HMGCR | Weighted Median | 6 | –1.694 | 0.275 | 7.30 × 10^–10^ | 0.184 | 0.107 | 0.315 | 0.107–0.315 |  |
|  | HMGCR | MR Egger | 6 | –1.380 | 1.269 | 0.338 | 0.252 | 0.021 | 3.027 | 0.021–3.027 |  |
|  | HMGCR | MR Presso | 6 | –1.597 | 0.133 | 6.96 × 10^–05^ | 0.202 | 0.156 | 0.263 | 0.156–0.263 |  |
|  | CETP | Inverse Variance Weighted | 4 | –2.063 | 0.331 | 1.60 × 10^–08^ | 0.127 | 0.066 | 0.243 | 0.066–0.243 |  |
|  | CETP | Weighted Median | 4 | –2.085 | 0.407 | 5.01 × 10^–06^ | 0.124 | 0.056 | 0.276 | 0.056–0.276 |  |
|  | CETP | MR Egger | 4 | –1.475 | 1.627 | 8.00 × 10^–03^ | 0.229 | 0.009 | 5.549 | 0.009–5.549 |  |
|  | CETP | MR Presso | 4 | –2.063 | 0.277 | 7.92 × 10^–05^ | 0.127 | 0.074 | 0.219 | 0.074–0.219 |  |
|  | APOC3 | Inverse Variance Weighted | 4 | –2.063 | 0.331 | 1.60 × 10^–08^ | 0.127 | 0.066 | 0.243 | 0.182–0.824 |  |
|  | APOC3 | Weighted Median | 4 | –2.085 | 0.407 | 5.01 × 10^–06^ | 0.124 | 0.056 | 0.276 | 0.182–1.016 |  |
|  | APOC3 | MR Egger | 4 | –1.475 | 1.627 | 8.00 × 10^–03^ | 0.229 | 0.009 | 5.549 | 0.001–14.0381 |  |
|  | APOC3 | MR Presso | 4 | –2.063 | 0.277 | 7.92 × 10^–05^ | 0.127 | 0.074 | 0.219 | 0.179–0.841 |  |
| TAA | PCSK9 | Inverse Variance Weighted | 13 | 0.021 | 0.100 | 4.42 × 10^–10^ | 1.021 | 0.840 | 1.241 | 0.840–1.241 |  |
|  | PCSK9 | Weighted Median | 13 | –0.093 | 0.121 | 3.03 × 10^–07^ | 0.911 | 0.719 | 1.155 | 0.719–1.155 |  |
|  | PCSK9 | MR Egger | 13 | –0.103 | 0.146 | 0.460 | 0.902 | 0.677 | 1.202 | 0.677–1.202 |  |
|  | PCSK9 | MR Presso | 13 | 0.021 | 0.084 | 0.005 | 1.021 | 0.866 | 1.204 | 0.866–1.204 |  |
|  | HMGCR | Inverse Variance Weighted | 6 | –0.587 | 0.205 | 0.004 | 0.556 | 0.372 | 0.831 | 0.372–0.831 |  |
|  | HMGCR | Weighted Median | 6 | –0.642 | 0.249 | 0.010 | 0.526 | 0.323 | 0.857 | 0.323–0.857 |  |
|  | HMGCR | MR Egger | 6 | –0.077 | 1.266 | 0.955 | 0.926 | 0.077 | 11.077 | 0.077–11.077 |  |
|  | HMGCR | MR Presso | 6 | –0.587 | 0.169 | 0.018 | 0.556 | 0.399 | 0.775 | 0.399–0.775 |  |
|  | CETP | Inverse Variance Weighted | 4 | 0.376 | 0.327 | 0.250 | 1.457 | 0.767 | 2.765 | 0.767–2.765 |  |
|  | CETP | Weighted Median | 4 | 0.369 | 0.384 | 0.336 | 1.447 | 0.682 | 3.070 | 0.682–3.070 |  |
|  | CETP | MR Egger | 4 | 0.258 | 1.601 | 0.887 | 1.295 | 0.056 | 29.859 | 0.056–29.859 |  |
|  | CETP | MR Presso | 4 | 0.376 | 0.215 | 0.179 | 1.457 | 0.956 | 2.220 | 0.956–2.220 |  |
|  | APOC3 | Inverse Variance Weighted | 4 | –0.257 | 0.358 | 0.473 | 0.773 | 0.384 | 1.559 | 0.384–1.559 |  |
|  | APOC3 | Weighted Median | 4 | –0.376 | 0.415 | 0.365 | 0.687 | 0.304 | 1.549 | 0.304–1.549 |  |
|  | APOC3 | MR Egger | 4 | –0.026 | 2.049 | 0.991 | 0.974 | 0.018 | 54.019 | 0.018–54.019 |  |
|  | APOC3 | MR Presso | 4 | –0.257 | 0.230 | 0.345 | 0.773 | 0.492 | 1.213 | 0.492–1.213 |  |
| AD | PCSK9 | Inverse Variance Weighted | 13 | 0.065 | 0.197 | 0.739 | 1.068 | 0.726 | 1.570 | 0.726–1.570 |  |
|  | PCSK9 | Weighted Median | 13 | –0.155 | 0.239 | 0.516 | 0.856 | 0.537 | 1.367 | 0.537–1.367 |  |
|  | PCSK9 | MR Egger | 13 | –0.406 | 0.289 | 0.187 | 0.666 | 0.378 | 1.173 | 0.378–1.173 |  |
|  | PCSK9 | MR Presso | 13 | 0.065 | 0.180 | 0.722 | 1.068 | 0.750 | 1.519 | 0.750–1.519 |  |
|  | HMGCR | Inverse Variance Weighted | 6 | –1.528 | 0.405 | 0.00016 | 0.217 | 0.098 | 0.480 | 0.098–0.480 |  |
|  | HMGCR | Weighted Median | 6 | –1.551 | 0.504 | 0.002 | 0.212 | 0.079 | 0.569 | 0.079–0.569 |  |
|  | HMGCR | MR Egger | 6 | 0.322 | 2.508 | 0.904 | 1.380 | 0.010 | 188.389 | 0.010–188.389 |  |
|  | HMGCR | MR Presso | 6 | –1.528 | 0.379 | 0.010 | 0.217 | 0.103 | 0.456 | 0.103–0.456 |  |
|  | CETP | Inverse Variance Weighted | 4 | 0.117 | 0.647 | 0.857 | 1.124 | 0.316 | 3.993 | 0.316–3.993 |  |
|  | CETP | Weighted Median | 4 | 0.290 | 0.751 | 0.699 | 1.337 | 0.306 | 5.830 | 0.306–5.830 |  |
|  | CETP | MR Egger | 4 | 0.840 | 3.167 | 0.816 | 2.316 | 0.005 | 1150.153 | 0.005–1150.153 |  |
|  | CETP | MR Presso | 4 | 0.117 | 0.395 | 0.787 | 1.124 | 0.518 | 2.439 | 0.518–2.439 |  |
|  | APOC3 | Inverse Variance Weighted | 4 | –0.221 | 0.707 | 0.754 | 0.801 | 0.200 | 3.204 | 0.200–3.204 |  |
|  | APOC3 | Weighted Median | 4 | –0.223 | 0.851 | 0.794 | 0.800 | 0.151 | 4.247 | 0.151–4.2466 |  |
|  | APOC3 | MR Egger | 4 | 1.417 | 4.553 | 0.785 | 4.124 | 0.001 | 30945.447 | 0.001–30945.447 |  |
|  | APOC3 | MR Presso | 4 | –0.221 | 0.671 | 0.763 | 0.802 | 0.215 | 2.988 | 0.215–2.988 |  |
| CAVS | PCSK9 | Inverse Variance Weighted | 13 | –0.332 | 0.062 | 9.28 × 10^–08^ | 0.717 | 0.635 | 0.810 | 0.635–0.810 |  |
|  | PCSK9 | Weighted Median | 13 | –0.317 | 0.077 | 3.43 × 10^–05^ | 0.728 | 0.627 | 0.846 | 0.627–0.846 |  |
|  | PCSK9 | MR Egger | 13 | –0.308 | 0.093 | 0.007 | 0.735 | 0.613 | 0.881 | 0.613–0.881 |  |
|  | PCSK9 | MR Presso | 13 | –0.332 | 0.027 | 3.73 × 10^–08^ | 0.717 | 0.680 | 0.756 | 0.680–0.756 |  |
|  | HMGCR | Inverse Variance Weighted | 6 | –0.591 | 0.125 | 2.27 × 10^–06^ | 0.554 | 0.433 | 0.707 | 0.433–0.707 |  |
|  | HMGCR | Weighted Median | 6 | –0.564 | 0.152 | 2.09 × 10^–04^ | 0.569 | 0.422 | 0.767 | 0.422–0.767 |  |
|  | HMGCR | MR Egger | 6 | 0.145 | 0.776 | 0.861 | 1.156 | 0.252 | 5.290 | 0.252–5.290 |  |
|  | HMGCR | MR Presso | 6 | –0.591 | 0.085 | 0.001 | 0.554 | 0.469 | 0.654 | 0.469–0.654 |  |
|  | CETP | Inverse Variance Weighted | 4 | 0.042 | 0.200 | 0.836 | 1.042 | 0.704 | 1.544 | 0.704–1.544 |  |
|  | CETP | Weighted Median | 4 | –0.045 | 0.225 | 0.842 | 0.956 | 0.615 | 1.486 | 0.615–1.486 |  |
|  | CETP | MR Egger | 4 | –0.870 | 0.981 | 0.469 | 0.419 | 0.061 | 2.866 | 0.061–2.866 |  |
|  | CETP | MR Presso | 4 | 0.042 | 0.115 | 0.742 | 1.043 | 0.832 | 1.367 | 0.832–1.167 |  |
|  | APOC3 | Inverse Variance Weighted | 4 | –0.617 | 0.219 | 0.005 | 0.540 | 0.351 | 0.829 | 0.351–0.829 |  |
|  | APOC3 | Weighted Median | 4 | –0.673 | 0.249 | 0.007 | 0.510 | 0.313 | 0.831 | 0.313–0.831 |  |
|  | APOC3 | MR Egger | 4 | –0.530 | 1.256 | 0.714 | 0.589 | 0.050 | 6.907 | 0.050–6.907 |  |
|  | APOC3 | MR Presso | 4 | –0.617 | 0.091 | 0.007 | 0.540 | 0.452 | 0.645 | 0.452–0.645 | 0.452–0.645 |

AAA, abdominal aortic aneurysm; AD, aortic dissection; APOC3, apolipoprotein C3; CAVS, calcific aortic valve stenosis; CETP, cholesteryl ester transfer protein; HMGCR, 3-hydroxy-3-methylglutaryl-coenzyme A reductase; PSCK9, proprotein convertase subtilisin/kexin type 9; # of SNPs, number of single-nucleotide polymorphisms; SD, standard deviation; TAA, thoracic aortic aneurysm.
